# Chilling or chemical induction of dormancy release in blackcurrant (*Ribes nigrum*) buds is associated with characteristic shifts in metabolite profiles

**DOI:** 10.1042/BCJ20240213

**Published:** 2024-08-09

**Authors:** Robert D. Hancock, Elisa Schulz, Susan R. Verrall, June Taylor, Michaël Méret, Rex M. Brennan, Gerard J. Bishop, Mark Else, Jerry V. Cross, Andrew J. Simkin

**Affiliations:** 1 Cell and Molecular Sciences, The James Hutton Institute, Invergowrie, Dundee DD2 5DA, U.K.; 2 MetaSysX GmbH, Am Mühlenberg 11, 14476 Potsdam-Golm, Germany; 3 Ecological Sciences, The James Hutton Institute, Invergowrie, Dundee DD2 5DA, U.K.; 4 NIAB, New Road, East Malling, Kent ME19 6BJ, U.K.; 5 NIAB, Huntingdon Road, Cambridge CB3 0LE, U.K.; 6 School of Life Sciences, University of Essex, Wivenhoe Park, Colchester CO4 3SQ, U.K.

**Keywords:** blackcurrant, bud dormancy, ERGER, winter chill

## Abstract

This study reveals striking differences in the content and composition of hydrophilic and lipophilic compounds in blackcurrant buds (*Ribes nigrum* L., cv. Ben Klibreck) resulting from winter chill or chemical dormancy release following treatment with ERGER, a biostimulant used to promote uniform bud break. Buds exposed to high winter chill exhibited widespread shifts in metabolite profiles relative to buds that experience winter chill by growth under plastic. Specifically, extensive chilling resulted in significant reductions in storage lipids and phospholipids, and increases in galactolipids relative to buds that experienced lower chill. Similarly, buds exposed to greater chill exhibited higher levels of many amino acids and dipeptides, and nucleotides and nucleotide phosphates than those exposed to lower chilling hours. Low chill buds (IN) subjected to ERGER treatment exhibited shifts in metabolite profiles similar to those resembling high chill buds that were evident as soon as 3 days after treatment. We hypothesise that chilling induces a metabolic shift which primes bud outgrowth by mobilising lipophilic energy reserves, enhancing phosphate availability by switching from membrane phospholipids to galactolipids and enhancing the availability of free amino acids for *de novo* protein synthesis by increasing protein turnover. Our results additionally suggest that ERGER acts at least in part by priming metabolism for bud outgrowth. Finally, the metabolic differences presented highlight the potential for developing biochemical markers for dormancy status providing an alternative to time-consuming forcing experiments.

## Introduction

Blackcurrant (*Ribes nigrum* L.) is a small, perennial shrub in the family *Grossulariaceae* native to temperate parts of central and Northern Europe and Northern Asia where it is widely cultivated both commercially and domestically [1]. The plant produces sweet, aromatic berries with a purple–black colour imparted by a range of anthocyanins, particularly delphinidin-3-*O*-glucoside, delphinidin-3-*O*-rutinoside, cyanidin-3-*O*-glucoside and cyanidin-3-*O*-rutinoside [2]. Along with a range of other polyphenolic compounds [3] and high concentrations of vitamin C [4], anthocyanins, found in many fruits [5–11], contribute to the antioxidant properties of blackcurrant fruit [10] that have been linked to the health benefits of soft fruit consumption. In the case of blackcurrants, they have been shown to have important pharmacological effects for the cardiovascular system [12–14], pulmonary system [15,16], nervous system [17] and reported anti-tumour activities [18–22].

During the winter, the buds of many temperate woody plants, including blackcurrant undergo a dormancy cycle to prevent premature bud burst during transient periods of warmer weather and subsequent shoot damage as colder weather returns. This cycle is induced by shortening day length and/or a decrease in temperature [23–25]. Following a period of adequate exposure to cold temperatures, dormancy is broken and bud growth can resume following a return to favourable growing conditions [26,27]. In the absence of sufficient chill, bud growth will not resume even when external conditions are suitable for its support. Furthermore, the sufficient winter chill is not always achieved and therefore the effective synchronisation of fruit ripening must be managed through the use of low chill cultivars, by techniques such as evaporative cooling, or by the application of dormancy break agents, which compensate for the lack of sufficiently low temperatures [28]. Global warming is further intensifying this problem in some areas by reducing the potential for sufficient winter chill to break bud dormancy [29] resulting in a further need to use dormancy break agents. Indeed, it has been suggested that some of the older yet still widely grown cultivars such as ‘Ben Lomond’ and ‘Ben Dorain’ may no longer achieve their chilling requirements under current U.K. climates [30].

Currently, there are many commercially available spray reagents on the market that promise a synchronised ripening of fruits when buds are treated. Bud break-enhancing compounds, such as hydrogen cyanamide, have also been linked to an increase in the number of flowers per shoot and a reduction in the number of side flowers [31–33]. One of these reagents, ERGER® is a biostimulant used to promote uniform bud break. It has been shown to break dormancy in both axillary and terminal buds in many species including apple and kiwifruit [34,35]. Hernández and Craig [35] also reported that following ERGER treatment, subsequent fruit quality was enhanced, with an increase in soluble solids content and dry matter possibly related to a change in the dates of bud break and flowering. ERGER is considered to be part of a new generation of products due to it being less harmful to the environment when compared with other commercially available products, (i.e. hydrogen cyanamide) whilst having broadly similar effect on bud dormancy [34].

Dormancy induction and release is a complex process driven by environmental factors such as photoperiod and temperature that is mediated via hormonal and other signalling pathways. Dormancy induction is promoted by ABA which acts via SHORT VEGETATIVE PHASE to induce callose synthase and restrict plasmodesmatal transport [36,37]. On the contrary, dormancy release is promoted by gibberellin-induced 1,3-β-glucanases, which degrade callose and re-establish symplastic connection [38]. Microarray and other evidence also indicate that other signalling and metabolic pathways, particularly sugar and reactive oxygen metabolism may further influence dormancy induction and release in species including blackcurrant [39–41]. While little is known regarding the mechanisms by which ERGER promotes bud dormancy release, a recent transcript study demonstrated that treatment consistently altered the expression of transcripts associated with redox processes and glucose metabolism. Furthermore, transcripts associated with gibberellin perception and several 1,3-β-glucanases were up-regulated by ERGER treatment while transcripts associated with ABA signalling were down-regulated [42].

In the present study, we aimed to complement previous transcriptomic studies regarding bud dormancy release in blackcurrant by conducting a study of metabolic changes associated with dormancy release. We hypothesised that a change in metabolic status would be associated with the switch from dormancy to bud growth that would be compatible with the provision of metabolites and energy required for bud growth. Furthermore, we aimed to gain a greater understanding of the mechanisms of action of ERGER by comparing metabolic changes in untreated versus ERGER-treated buds. Our hypothesis was that downstream of dormancy release, the same mechanisms of bud development would apply hence ERGER or chilling-induced dormancy release would be associated with similar metabolic profiles.

## Results

**Figure 1.**
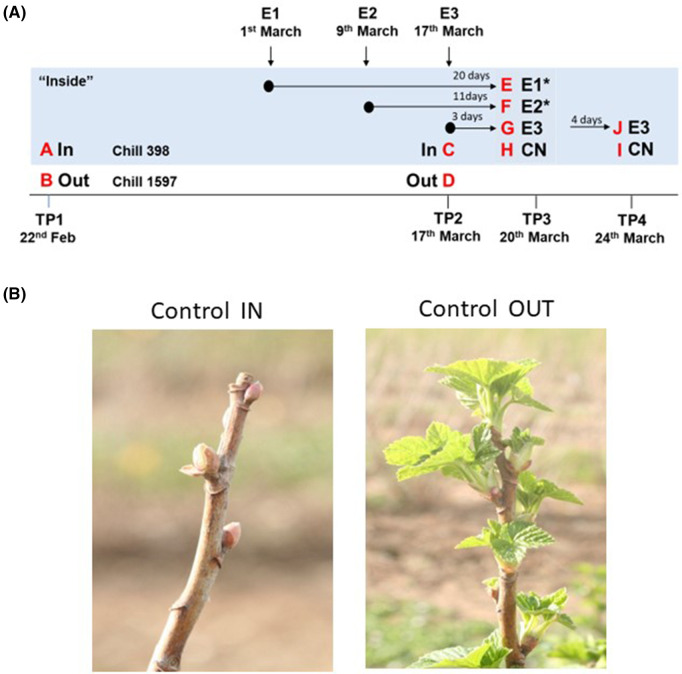
Bud break in blackcurrant cultivated at New House Farm, Canterbury, Kent, U.K. in 2017. (**A**) Experimental dates and time points of ERGER treatments (E1–E3) on the 1, 9 and 17 March 2017 (see arrows) for IN samples. (A–J) Letters denoted time points that samples were collected on the 22 February (Time Point 1 (TP1), 17 March (TP2), 20 March (TP3) and 24 March (TP4)) 2017. (**B**) Image of bud break in IN and OUT samples on the 30 March 2017. * = buds collected were dormant but buds on plants were breaking.

Buds were harvested from plants left in the open field ‘Out’ or under cover ‘In’ on the 22 February (TP1). Data loggers indicated that ‘Out’ plants had received a total of 1597 chill hours (below 7°C) while ‘In’ plants had received a total of 398 chill hours. This compares with a requirement of ∼3000 h chilling to achieve complete bud burst in intact cuttings of several cultivars grown in the U.K. [43]. Untreated plants grown under ‘In’ or ‘Out’ conditions were again harvested on 17 March (Figure 1A) and the dormancy status of plants was estimated by visual inspection on March 30 (Figure 1B), which clearly indicated that dormancy break was severely retarded in plants grown under the warmer ‘In’ conditions.

To test the effect of the ERGER on bud burst and metabolism, low chill ‘In’ plants were treated according to the manufacturer's instructions on the 1 March (E1), 9 March (E2), or the 17 March (E3) and bud samples were collected on March 20. This represented 20 days (E1), 11 days (E2) and 3 days (E3) after treatment alongside a contemporary untreated sample (CN; see Figure 1A).

### The majority of metabolite features are common across bud dormancy stages

A total of 4534 unique chromatographic features were detected using a combination of GC/MS and positive and negative ion mode LC/MS for hydrophilic and lipophilic fractions, respectively. A total of 651 features were annotated (Supplementary Table S1) comprising a range of primary (sugars, organic acids, amino acids, fatty acids, complex lipids) and secondary (primarily polyphenols) metabolites. Several of the annotated features were identified in multiple analytical runs.

An analysis of the presence or absence of features in buds collected across the transition from dormancy to activity indicated that of the 4534 features, 4130 were found in all samples (Supplementary Figure S1A). Similarly, when ‘In’ and ‘Out’ bud samples collected at TP1 and TP2 were compared, ∼4200 features were common between samples (Supplementary Figure S1B). Many of the unique features in these comparisons were close to the limits of detection and hence the absence of specific features in specific samples may simply reflect an abundance below the limit of detection where features are apparently absent.

**Figure 2.**
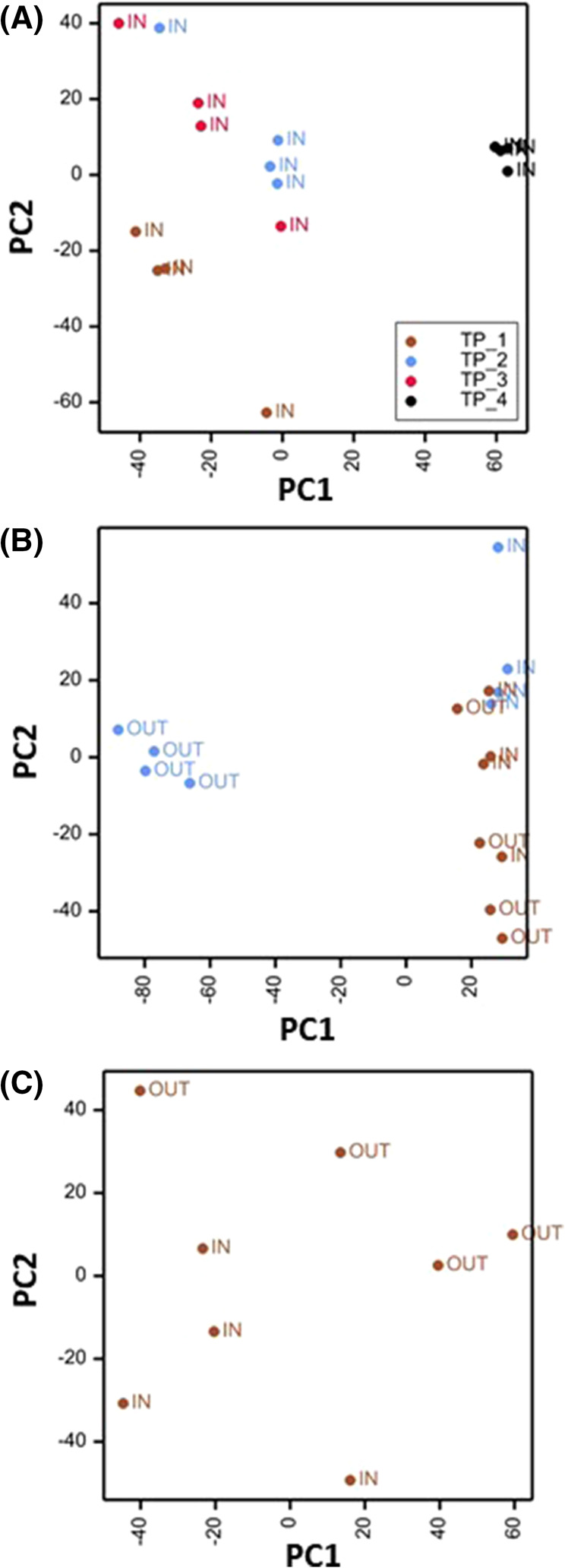
PCA plots of normalised data from bud samples collected at four separate stages of dormancy release. (**A**) ‘In’ samples collected on 22 February (TP_1), 17 March (TP_2), 20 March (TP_3) or 24 March (TP_4). (**B**) ‘In’ and ‘Out’ samples collected on 22 February (TP_1) or 17 March (TP_2). (**C**) ‘In’ and ‘Out’ samples collected on 22 February (TP_1).

To gain a greater insight into metabolite differences underpinning different states of bud dormancy, principal components analysis (PCA) was conducted on metabolite profiles of ‘In’ buds collected at four different timepoints. As indicated in Figure 2A, buds collected on 22 February (TP1) were clearly separated from those collected on the 17 and 20 March (TP2, 3) which were again clearly separated from those collected on 24 March (TP4) after visible signs of bud burst were observed. The majority of separation was attributable to component 1 representing almost 50% of the variance although there was also some separation on component 2. Replicates, particularly those collected at TP4 were closely clustered. These data suggest a major shift in metabolite profiles between ecodormant and inactive buds (TP1), buds undergoing early non-visible bud burst (TP2, 3) and those undergoing visible development (TP4).

A similar comparison of ‘In’ and ‘Out’ buds at TP1 and 2 indicated a clear separation of ‘Out’ buds at TP2, driven by principal component 1 representing >60% of the variance (Figure 2B). ‘In’ buds at TP1 and TP2 were closely clustered and were partially separated from TP1 ‘Out’ buds on PC2 and this separation was more evident when ‘In’ and ‘Out’ TP1 samples were analysed together in the absence of other samples (Figure 2C). Given that ‘In’ samples had only received <400 h of chilling at TP1 (relative to 1600 h for ‘Out’ samples) and that even after exposure to permissive conditions for growth, no bud burst was apparent (Figure 1B), we hypothesise that ‘In’ samples remained endodormant. The clear differences between TP2 ‘Out’ and other samples suggest changes in metabolism prior to the visible growth of ecodormant buds while the clear differences between TP1 ‘In’ and ‘Out’ samples suggest differences in metabolite profiles between endodormant and ecodormant buds.

### Bud activity is associated with a significant shift in bud metabolism

To understand the specific metabolic changes associated with bud activity, we focussed on changes occurring in annotated metabolites, particularly membrane associated and storage lipids, and primary metabolites including those present in the interlinked pathways of sugar, organic acid and amino acid metabolism.

#### Bud activity results in a massive restructuring of lipid profiles

**Figure 3.**
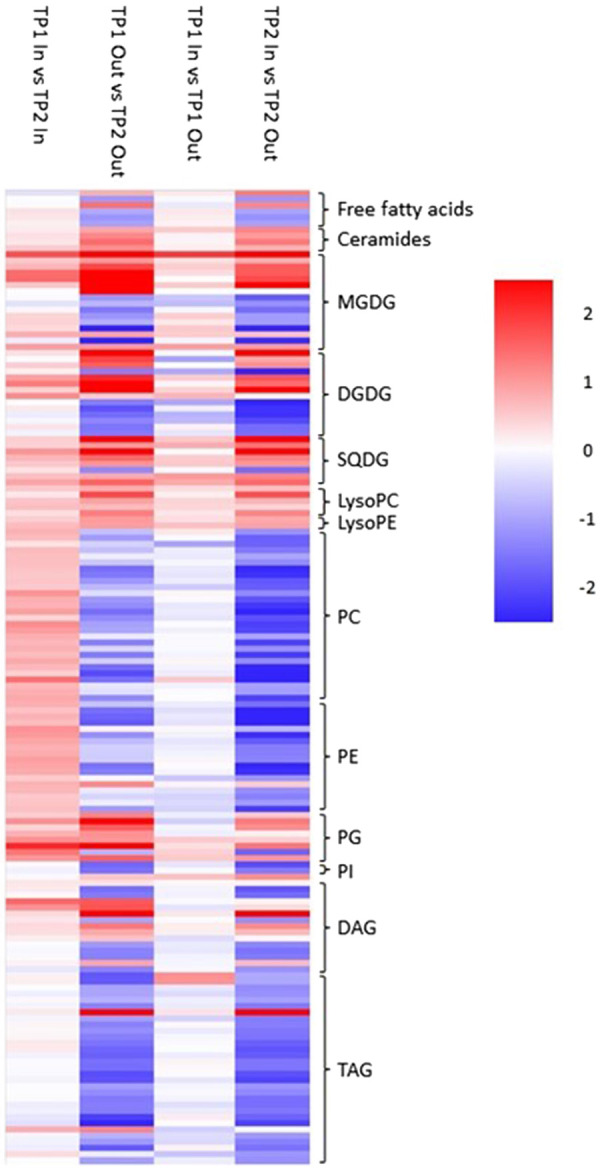
Relative fold change in lipids between ‘In’ and ‘Out’ blackcurrant buds sampled at different times. Heatmap represents log_2_ fold change in relative lipid content between samples as indicated by column headings. Lipid classes are represented according to the brackets indicated and the magnitude of the change is represented by the scale bar. MGDG, monogalactosyl diacylglycerol; DGDG, diglactosyl diacylglycerol; SQDG, sulphoquinovosyl diacylglycerol; LysoPC, (lyso)phosphatidylcholine; LysoPE, (lyso)phosphatidylethanolamine; PG, phosphatidylglycerol; PI, phosphatidylinositol; DAG, diacylglycerol; TAG, triacylglycerol.

Lipid profiles were massively altered in ‘Out’ buds between TP1 and TP2 (Figure 3). Large numbers of di- and triacylglycerol (DAG and TAG) lipids were strongly reduced suggesting mobilisation of storage compounds in anticipation of tissue replication and growth. More surprisingly, large numbers of phospholipids were strongly reduced in the buds sampled at TP2, particularly phosphatidylcholines and ethanolamines that are the dominant lipids in membranes [44]. This was accompanied with large shifts in the level of plastid synthesised galactolipids [45,46], some of which increased while others decreased. On the contrary, the majority of plastid membrane-associated sulphoquinovosyl lipids increased [47].

Changes in lipid content between ‘In’ samples from the same timepoints were not as extensive as those for ‘Out’ samples, as might be expected from comparison of two samples that were both considered to be endodormant due to insufficient winter chill (Figure 3). Furthermore, changes in the amounts of individual lipid species were often the opposite of those seen ‘Out’ buds. For example, levels of phospholipids mostly increased.

Comparison of ‘In’ with ‘Out’ samples from the same timepoint provided further evidence of massive changes in lipid profiles following a shift to bud activity where a comparison of endordormant ‘In’ and active ‘Out’ samples from TP2 showed a similar change in lipid profiles to ‘Out’ samples at TP2 relative to TP1. Interestingly, a comparison of ‘In’ and ‘Out’ samples at TP1 also showed a similar but significantly weaker change in lipid profiles to ecodormant and active ‘Out’ samples suggesting that change in lipid profiles may precede committed metabolic activity for bud outgrowth and could act as potential markers for bud dormancy status.

Taken together our data suggest that bud activity requires massive changes in bud lipid profiles. In agreement with previous reports [48], we observed a turnover of storage lipids to provide the carbon building blocks and reducing power for tissue growth. Similarly, it has previously been reported that phospholipids can act as a storage reserve for phosphate in *Populus *× *canescens* [48] and we, therefore, propose that in blackcurrant buds membrane phospholipids are turned over to provide phosphate for synthesis of nucleic acids. This requires a restructuring of membranes leading to alterations in galactolipid profiles. Finally, the biogenesis of proplastids in anticipation of photosynthesis results in an increase in bud sulphoquinovosyl lipids.

#### Bud activation promotes primary metabolism

**Figure 4.**
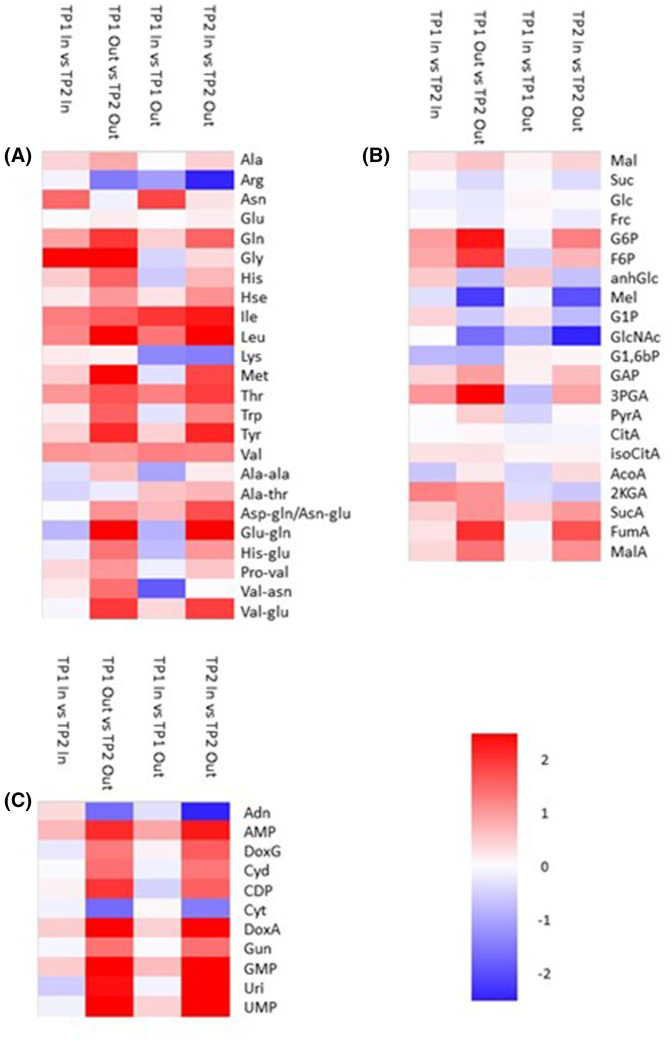
Relative fold change in a range of metabolites between ‘In’ and ‘Out’ blackcurrant buds sampled at different times. Heatmaps indicate fold changes (log_2_, according to the scale shown) in amino acids (**A**), sugars and organic acids (**B**), and nucleotides and nucleotide phosphates (**C**) between different bud samples as indicated by column headings. Legends to the right of rows indicate individual compounds with amino acids and dipeptides (**A**) represented according to the standard three letter code. Sugars (**B**) are represented as Mal, maltose; Suc, sucrose; Glc, glucose; Frc, fructose; G6P, glucose-6-P; F6P, fructose-6-P; anhGlc, anhydroglucose; G1P, glucose-1-P; GlcNAC, *N*-acetyl glucosamine; G1,6bP, glucose-1,6-bisphosphate; GAP, glyceraldehyde-3-phosphate; 3PGA, 3-phosphoglycerate; PyrA, pyruvate; CitA, citrate; isoCitA, isocitrate; AcoA, aconitate; 2KGA, 2-ketoglutarate; SucA, succinate; FumA, fumare; MalA, malate. Nucleotides (C) are abbreviated as Adn, adenosine; AMP, adenosine monophosphate; DoxG, deoxyguanosine; Cyd, cytidine; CDP, cytidine diphosphate; Cyt, cytosine; DoxA, deoxyadenine; Gun, guanosine; GMP, guanosine monophosphate; Uri, uridine; UMP, uridine monophosphate.

The reduction in storage lipids on bud activation prompted us to investigate how levels of primary metabolites changed upon bud activation. There was a clear increase in the content of almost all amino acids and dipeptides in ‘Out’ buds between TP1 and TP2 and a similar pattern was observed when comparing inactive ‘In’ buds with active ‘Out’ buds at TP2 (Figure 4A). As was observed for complex lipids, changes in free amino acids were much lower when comparing ‘In’ buds at TP1 and TP2 and while there was a moderate rise in some amino acids, many others exhibited a lower content in ‘In’ buds harvested at TP2. Interestingly, when comparing ‘In’ and ‘Out’ buds at TP1 there was again no clear overall rise in free amino acids and dipeptides (Figure 4A). This is in contrast with the situation observed in complex lipids where the differences between ‘In’ and ‘Out’ buds resembled those between ‘Out’ buds at TP1 and TP2 although at a lower magnitude. These data suggest either that lipid remodelling may be a marker of endodormancy release or that lipid remodelling precedes protein remodelling as buds move from dormancy to activity.

Increases in free amino acids have been observed in leaves under conditions of nitrogen deficiency [49] or undergoing natural senescence [50] where proteins are turned over either to allow remodelling in response to nutrient limitation or for export to seed. Similar, observations have been observed during the early stages of development in sweet cherry buds [51,52] where amino acids accumulated at the earliest stages of ontogenetic development prior to increases in bud total N content. It thus appears that a rise in free amino acids is frequently related to protein turnover and remodelling to meet changing requirements under the earliest stages of bud development. We, therefore, interpret the rise in free amino acids as a turnover of proteins to allow the resumption of bud growth.

Analysis of sugars, glycolytic and TCA cycle metabolites suggested an activation of primary catabolic pathways upon bud activation. Compared with buds collected at TP1, ‘Out’ buds collected at TP2 had slightly lower glucose, fructose and sucrose but marginally higher contents of maltose (Figure 4B). These data suggest that upon activation buds catabolise primary metabolic sugars whilst the increased maltose content could be indicative of starch breakdown to maintain sugar supply. This hypothesis was supported by the observation that many glycolytic and TCA cycle intermediates such as fructose-6-phosphate, gyceraldehyde-3-phosphate, succinate and malate were elevated in ‘Out’ buds relative to ‘In’ buds at TP1, and particularly at TP2 (Figure 4B).

As was observed for amino acids, a comparison of ‘In’ and ‘Out’ buds at TP1 or of ‘In’ buds at TP2 compared with TP1 revealed much smaller differences in sugars, glycolytic intermediates and TCA cycle metabolites (Figure 4B). These data suggest that changes in primary metabolic pathways occur after lipid remodelling, are dependent on bud activity and are poor markers of bud dormancy status.

In support of the hypothesis that phospholipids act as a phosphate store to be utilised at bud activation, we observed a major increase in free nucleotides and nucleotide phosphates when comparing inactive buds (‘In’ buds at TP2, ‘Out’ buds at TP1) with active (‘Out’ buds TP2) buds (Figure 4C), perhaps indicative of a need for nucleic acid synthesis during rapid cell division at the early stages of bud activity. Again, this pattern was not observed when comparing TP2 and TP1 ‘In’ buds or ‘In’ and ‘Out’ buds at TP1.

### ERGER treatment promotes metabolic adjustments consistent with the activation of dormant buds

The metabolomic comparison of ‘In’ and ‘Out’ buds described above suggests that despite being in a warmer environment than ‘Out’ buds, ‘In’ buds remained inactive at least until March 17, probably as result of receiving insufficient winter chill to overcome endo- or paradormancy. To determine the impact of the dormancy-breaking treatment ERGER on the bud metabolome, ‘In’ buds were treated with ERGER as described and then harvested on the same date 20-, 11- or 3-days post treatment (TP3 E1, E2, E3, respectively) or in the case of the E3 treatment again on the 24 March TP4 E3, 7 days after ERGER treatment.

**Figure 5.**
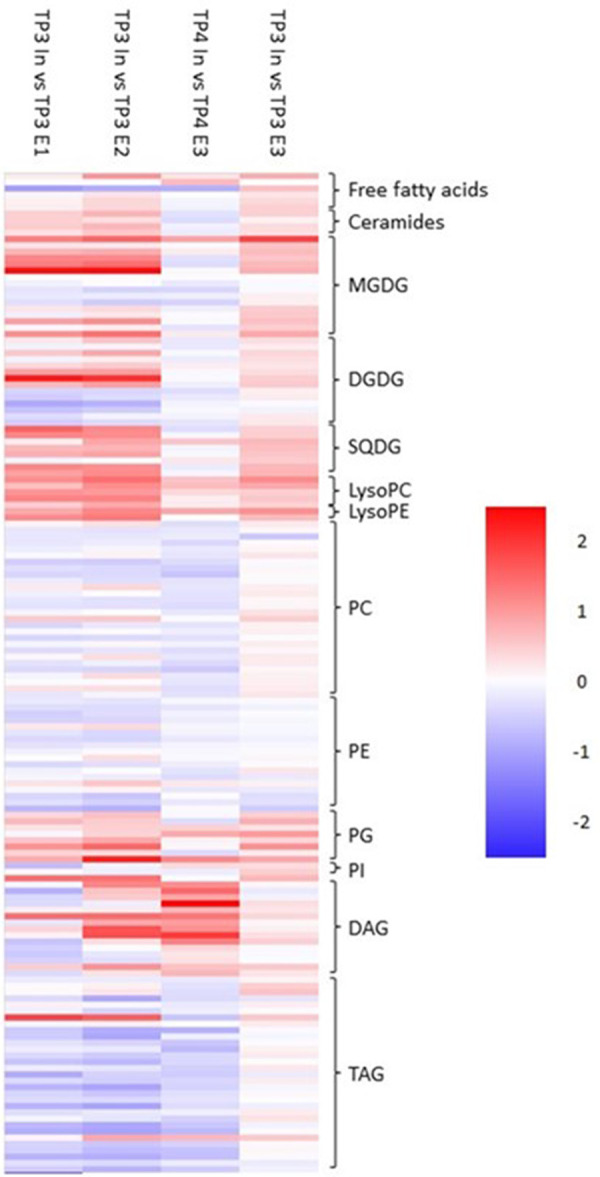
Relative fold change in lipids in control and ERGER-treated ‘In’ buds at different times following ERGER treatment. Heatmap represents log_2_ fold change in relative lipid content between control and ERGER-treated buds sampled 20 (TP3 In vs TP3 E1), 11 (TP3 vs TP3 E2), 7 (TP4 In vs TP4 E3) or 3 days (TP3 In vs TP3 E3) after treatment. Lipid classes are represented according to the brackets indicated and the magnitude of the change is represented by the scale bar. MGDG, monogalactosyl diacylglycerol; DGDG, diglactosyl diacylglycerol; SQDG, sulphoquinovosyl diacylglycerol; LysoPC, (lyso)phosphatidylcholine; LysoPE, (lyso)phosphatidylethanolamine; PG, phosphatidylglycerol; PI, phosphatidylinositol; DAG, diacylglycerol; TAG, triacylglycerol.

Comparison of T3 ‘In’ buds with TP3 E3 ‘In’ buds that were treated with ERGER 3 days prior to harvest revealed minor changes in lipid profiles where the majority of lipids were marginally higher in ERGER-treated relative to control buds (Figure 5). Seven days after treatment, more distinct changes in lipid were observed where phosphatidylcholines and ethanolamines were lower in treated that control buds. Similarly, TAGs were reduced while DAGs were increased, perhaps suggestive of early storage lipid mobilisation. Eleven days after ERGER treatment, the relative content of many galactolipids began to increase while the reduction in phospholipids and TAGs became more evident (Figure 5). At 20 days after treatment, many DAGs also declined relative to control buds with the pattern of changes in lipid content becoming increasingly similar to that observed on the transition from bud dormancy to activity in chilled buds (Figure 5 cf. Figure 3).

**Figure 6.**
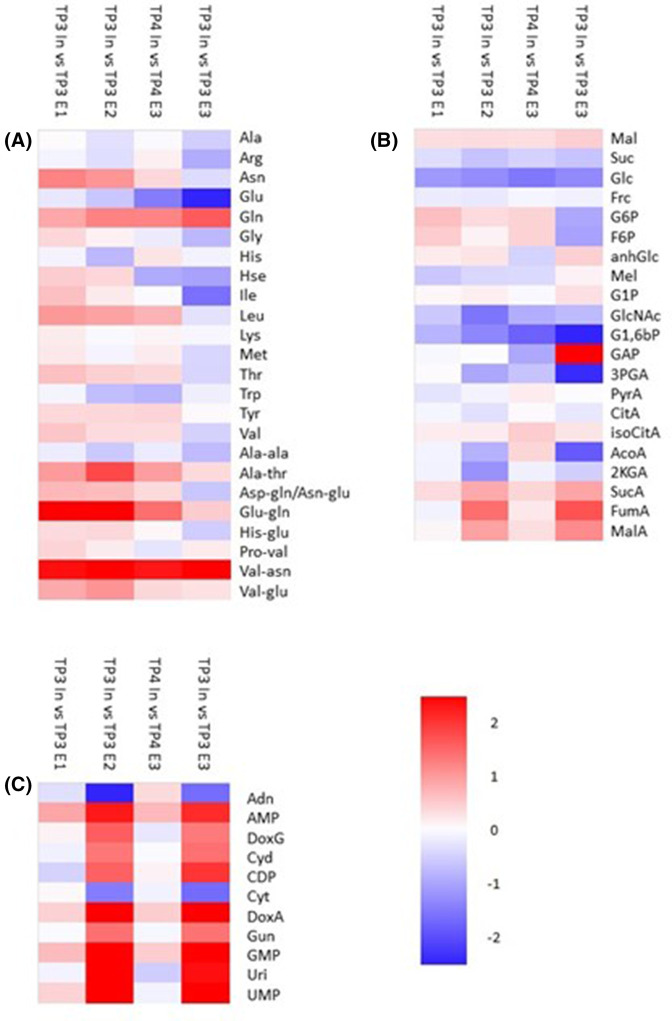
Relative fold change in polar metabolites in control and ERGER-treated ‘In’ buds at different times following ERGER treatment. Heatmap represents log_2_ fold change in relative content of amino acids and dipeptides (**A**), sugars and organic acids (**B**), and nucleotides and nucleotide phosphates (**C**) between control and ERGER-treated buds sampled 20 (TP3 In vs TP3 E1), 11 (TP3 vs TP3 E2), 7 (TP4 In vs TP4 E3) or 3 days (TP3 In vs TP3 E3) after treatment. Legends to the right of rows indicate individual compounds with amino acids and dipeptides (**A**) represented according to the standard three-letter code. Sugars (**B**) are represented as Mal, maltose; Suc, sucrose; Glc, glucose; Frc, fructose; G6P, glucose-6-P; F6P, fructose-6-P; anhGlc, anhydroglucose; G1P, glucose-1-P; GlcNAC, *N*-acetyl glucosamine; G1,6bP, glucose-1,6-bisphosphate; GAP, glyceraldehyde-3-phosphate; 3PGA, 3-phosphoglycerate; PyrA, pyruvate; CitA, citrate; isoCitA, isocitrate; AcoA, aconitate; 2KGA, 2-ketoglutarate; SucA, succinate; FumA, fumarate; MalA, malate. Nucleotides (**C**) are abbreviated as Adn, adenosine; AMP, adenosine monophosphate; DoxG, deoxyguanosine; Cyd, cytidine; CDP, cytidine diphosphate; Cyt, cytosine; DoxA, deoxyadenine; Gun, guanosine; GMP, guanosine monophosphate; Uri, uridine; UMP, uridine monophosphate.

Three days after ERGER treatment, the majority of amino acids and dipeptides were slightly reduced; however, by 7 days many began to increase and after 20 days the pattern of accumulation closely resembled that of active buds (TP1 ‘Out’ vs TP2 ‘Out’; Figures 4A and 6A). Patterns of accumulation of sugars, glycolytic and TCA cycle intermediates varied with time after ERGER treatment (Figure 6B**)** although there were some similarities to the changes observed on the transition from bud dormancy to activity (Figure 4B). Changes in nucleotides and nucleotide phosphates were similarly dynamic with increases after 3- and 11-days treatment although changes between control and ERGER-treated buds were less apparent at 7- and 20-days post treatment (Figure 6B).

## Discussion

Bud dormancy induction is a complex developmental process dependent on the perception of external and endogenous cues centred around daylength perception and integration with circadian signalling [53]. Dormancy is maintained by regulation of the balance between ABA and GA signalling which is integrated via the activity of the MADS-box gene SHORT VEGETATIVE PHASE LIKE (SVL). SVL expression is in turn regulated by EARLY BUD BREAK 1 (EBB1) and temperature-dependent EBB3 ultimately resulting in dormancy release and bud break [54].

Despite the significant advances made in recent years with respect to understanding the molecular players underpinning bud dormancy induction and release, the metabolic shifts associated with bud dormancy release have not been widely examined with only a few studies available in key soft crops such as blueberry and blackcurrant [55,56]. Our research, therefore, provides new insights into the molecular adjustments associated with dormancy release as outlined below.

### Di- and Triacylglycerols are mobilised upon bud break to provide ‘fuel’ for growth

Following a sufficient period of winter chill, DAGs and TAGs decrease in abundance when dormant buds are compared with active buds. DAG and TAG function as storage lipids, which can be broken down to provide energy for growth and development [57]. Oils in the form of TAG are abundant in tissues such as seeds where they form the main component of vegetable oils and act as an energy source for germination. The data presented in this manuscript show that upon the release of blackcurrant dormancy, DAG and TAG levels decrease. As plants switch to vegetative growth, DAG and TAG are mobilised to provide ‘fuel’ for bud growth. Interestingly, these changes in DAG and TAG can be induced by the application of ERGER. Following treatment, a decrease in TAG was initially observed within 7 days of treatment associated with an increase in DAG. Twenty days after treatment, both TAG and many DAG had decreased with a profile similar to that of chilled buds. These data suggest sequential removal of acyl chains initially to form DAG from TAG followed by further acetylesterase activity to release additional fatty acid chains as the storage lipid pool becomes more diminished. Similar results have previously been observed in poplar and beech trees suggesting the strategy may be common to woody perennials [48].

### Phospholipids act as a potential phosphate pool

Phospholipids are a structurally diverse set of lipid species, including phosphatidylcholine (PC), phosphatidylethanolamine (PE), phosphatidylglycerol (PG), phosphatidylserine (PS) and phosphoinositides (PI) [58]. Phospholipids are key components of cell membranes that form the outer envelope of the cell, internal cellular compartments (e.g. vacuole), plasma membrane, *trans*-Golgi network (TGN) and Golgi, and are also present in the membranes of subcellular organelles such as chloroplasts and mitochondria and have been noted to accumulate as a pool of phospholipids in the nucleus where they regulate nuclear function [59].

As growth resumes following dormancy release, increased phosphate demand might be expected for DNA and RNA synthesis as well as energy transfer. We observed a large reduction in the majority of PCs and PEs as chilling hours increased accompanied by an increase in nucleotides and nucleotide phosphates which may be indicative of the mobilisation of phosphate from lipid pools for the synthesis of nucleic acids. Similar observations were observed following treatment with ERGER suggesting that ERGER triggers parallel metabolic changes to those caused by chilling.

### Bud break triggers the formation and mobilisation of key galactolipids involved in chloroplast development

Galactolipids, monogalactosyldiacylglycerol (MGDG) and digalactosyldiacylglycerol (DGDG) are synthesised in plastids (chloroplasts) and are the predominant lipid in the thylakoid membrane [60]. They are also found associated with other cellular membranes where they can substitute for phospholipids to conserve phosphate for other essential processes.

Chloroplast membranes contain a high quantity of MGDG and DGDG, essential for the synthesis of a functional photosynthetic apparatus, accounting for ∼50% and 25% of total thylakoid lipids, respectively [45,61,62]. In Arabidopsis mutants with a reduction in the accumulation of DGDGs, the photosynthetic performance is impaired [63] and impaired PSI, weakening the flux of electrons through the photosynthetic electron chain [64]. Furthermore, mutants deficient in the accumulation of MGDGs show impaired formation and maintenance of both PSI and PSII photosynthetic apparatus [65,66]. Following winter chill, increases in some MGDG and DGDG galactolipids are consistent with the plants undergoing bud initiation, accompanied by chloroplast biogenesis and greening of the tissue (i.e. increases in chlorophyll) for photosynthesis. SQDG, sulfur-containing anionic glycerolipid, is mostly associated with chloroplast membranes [67,68]; however, its proportion increases upon Pi starvation, due to its ability to substitute for the phospholipid PG [69,70], which may be in response to a decrease in phospholipids and remobilisation of phosphate for growth. A comparison of ERGER-treated buds and control buds at TP3 reveals a similar but weaker metabolic profile to those shown by active versus dormant buds.

### Amino acids increase following bud break

Amino acids are formed by nitrogen assimilation to produce the primary amino acids glutamate, glutamine, aspartate and asparagine. In addition to their use during protein biosynthesis, they have many other important functions in plants, including playing key roles in signalling responses in response to plant stresses as well as being precursors for the formation of phytohormones and secondary metabolites [71–74]. Following the initiation of bud break, an increase in a palate of amino acids is observed. In Arabidopsis, during germination, which occurs in the absence of light, the breakdown of seed storage proteins provides the amino acids necessary for growth and the production of proteins required for photosynthesis [75]. This suggests that the increase in free amino acids observed is either due to an increase in amino acid import, the degradation of storage proteins or the remobilisation of proteins associated with dormancy. Previous works have shown that proteins constitute a store of amino acids that can be remobilized; these amino acids are subsequently recycled and allocated to provide the building blocks for proteins required for growth. ERGER treatment also results in an increase in amino acids; however, these increases are weaker than those observed due to cold-induced bud break suggesting the additional signals due to cold exposure may be behind this rapid increase in amino acids, and that ERGER may not replicate all mechanisms of natural bud break.

### Carbohydrates are rapidly metabolised after bud-break

Carbohydrates are the primary products of photosynthesis and act as the primary building blocks for all plant metabolites. In addition to being a key source of carbon and energy for growth, sugars regulate cell division, cell differentiation and cell growth [76–79]. Previous works have reported that sugars can be rapidly redistributed, often over considerable distances and accumulate in auxiliary buds correlating in dormancy release after shoot tip removal [80,81]. Radchuk et al. [80] also reported that artificially increasing sucrose levels suppresses the expression of the transcription factor BRANCHED1 (BRC1), involved in maintaining bud dormancy, resulting in rapid bud break in pea. In blackcurrant, following bud-break, several sugars including fructose, d-galactose and d-ribose were depleted. These sugars provide fuel for bud growth and in the case of d-ribose, it is used for the creation of ATP and is the backbone for RNA and DNA. d-Galactose readily converted to glucose, which is more easily assimilated and can be converted to ascorbic and d-galacturonic acids. d-Galactose is a precursor to glucose production and is an important energy-providing sugar. Interestingly, the decrease in d-galactose is accompanied by an increase in d-galacturonic acids suggesting that d-galactose is being metabolised during bud break. Interestingly, sucrose levels remained relatively constant, a slight decrease may be seen; however, the increase in sucrose triggering bud release, as seen in pea is not observed. However, we cannot rule out that a rapid increase in sucrose triggers rapid dormancy release before that sucrose is quickly depleted as plant metabolism increases. However, following ERGER treatment, these decreases are more difficult to see, although a similar yet weaker pattern of sugar turnover is apparent at 11 days post treatment.

### Metabolites as markers of bud status

Our data clearly demonstrate large shifts in both lipophilic and polar metabolites on transition from bud dormancy to activity. In particular, there are large and consistent shifts in galactolipids, phospholipids and storage lipids on the transition from dormant to active status induced either by the accumulation of sufficient winter chill or by the application of the dormancy-breaking agent ERGER. Similarly, a consistent change in free amino acids was observed as early as 3 days following ERGER treatment. Currently, studies on dormancy in perennial crops are limited by the need to define dormancy status by placing whole plants, isolated branches or isolated nodes into a forcing environment, typically for 2 weeks (e.g. [40]). Our data present the opportunity to define bud dormancy status within 24 h by conducting a metabolic analysis. This has the potential to allow much greater integration of physiological, molecular and biochemical approaches than is currently feasible thereby offering opportunities to significantly advance bud dormancy research.

## Conclusion

In the present manuscript, we have compared metabolite profiles of blackcurrant buds that have received sufficient winter chill to break bud dormancy (‘Out’ buds) with those that did not receive sufficient chill (In) buds during the transition from bud ecodormancy to bud activation.

Our data indicate that bud activation is associated with a strictly regulated reprogramming of the metabolism. These data suggest that blackcurrant bud activation is associated with (i) the mobilisation of storage lipids (DAG, TAG) to provide the ‘fuel’ for bud growth, (ii) a shift in the organisation of membrane lipids to release phosphate required for growth, (iii) increased flux through catabolic pathways (glycolysis and TCA cycle) to provide the energy and reducing power required for growth as well as important biosynthetic scaffolds, (iv) an increase in free amino acids suggesting either an increase in amino acid import, the degradation of storage proteins or the remobilisation of proteins associated with dormancy to provide the building blocks for proteins required for growth and (v) an increase in free nucleotides and nucleotide phosphates potentially associated with the synthesis of nucleic acids during cell division. These metabolic changes provide a series of potential markers that could be used to determine the dormancy status of the bud.

A comparison of inactive ecodormant buds to inactive para- or endodormant buds (TP1 ‘In’ vs ‘Out’) suggests that lipid restructuring, particularly turnover of storage and phospholipids may occur prior to bud activation and other metabolic adjustments indicating that changes in lipid profile could act as a potential marker of bud dormancy status providing a significant improvement on current methods of assessment which typically require scoring of bud growth 2 weeks after transfer to a forcing environment.

Treatment of inactive dormant buds with ERGER reveals a shift in lipid profile to those shown by active buds within 7 days after treatment. Furthermore, in an environment permissive for bud outgrowth other changes in primary metabolism consistent with bud activation such as the accumulation of amino acids are observed but at slightly later times. These data reinforce the suggestion that lipid profiles may act as a marker of bud dormancy status in blackcurrant and suggest that while changes in lipid profiles may be associated with dormancy release, changes in other primary metabolic pathways lie downstream and are associated with a shift to active growth resumption. Future work will seek to identify specific markers that can be used in industry to determine the status of bud break and therefore the requirement for treatment with biostimulants such as ERGER.

## Materials and methods

### Plant material and experimental design

Blackcurrant plants cv. Ben Klibreck, described as having moderate to high chilling requirements [30] (www.huttonltd.com/services/plant-varieties-breeding-licensing/blackcurrant/ben-klibreck), were grown in the field close to Canterbury, Kent, U.K. (51°15′04″N, 0°55′29″E) under standard industry management conditions. Six-year-old plants were used in all experiments. Plants were allowed to enter dormancy under field conditions and to manipulate winter chill, half of the plants were covered under three layers of horticultural plastic on December 1. ‘ERGER’, a mix of ERGER® and Active ERGER®, was applied with a motorised air-assisted backpack sprayer at a volume rate of 400 L/Ha, the concentration of ERGER® was 5L/HL, the rate/Ha was 20L, the concentration of Active ERGER® was 7.5L/HL, the rate/Ha 30L. There were no wetting agents and the controls were not sprayed.

### Bud sampling and extraction

Buds were sampled from four replicate plots each comprising three plants. At each timepoint, buds were removed with a sharp scalpel and directly transferred to liquid nitrogen prior to storage at −80°C. Frozen samples were prepared and metabolites and lipids were extracted by one-step extraction method as previously described [82].

### Metabolomics analysis

Metabolomics and lipidomics analyses were performed at metaSysX GmbH. Metabolite profiles were collected using a combination of LC/ and GC/MS. LC/MS was conducted using a Waters ACQUITY Ultra Performance Liquid Chromatograph (RP-UPLC) coupled to a Thermo-Fisher Exactive mass spectrometer comprising an ElectroSpray Ionisation source (ESI) and an Orbitrap mass analyzer (UPLC–MS). GC/MS was conducted using an Agilent Technologies Gas Chromatography (GC) coupled to a Leco Pegasus HT mass spectrometer which consists of an Electron Impact ionisation source (EI) and a time of flight (TOF) mass analyzer (GC–MS).

### LC–MS measurements (hydrophilic and lipophilic analysis), data processing and annotation

Samples were separated on a C18 reverse phase column (100 × 2.1 mm i.d. 1.8 μm particle size, Waters) at a temperature of 40°C. The mobile phases consisted of 0.1% formic acid in water (Solvent A) and 0.1% formic acid in acetonitrile (Solvent B). The flow rate of the mobile phase was 400 μl/min, and 2 μl samples were loaded per injection. Gradient conditions were: 0−1 min hold at 1% B, 1−13 min linear gradient 1%−35% B, 13−14.5 min linear gradient from 35%−70% B, 14.5−15.5 min linear gradient 70%−99% B, 15.5−17 min hold at 99% B, 17−17.5 min linear gradient to 1% B and 17.5−20 min hold at 1% B [83]. Mass spectra were acquired in full scan MS positive and negative modes (Mass Range [100−1500]). Extraction of the LC–MS data was accomplished with the software REFINER MS® 10.5 (GeneData, http://www.genedata.com). After extraction of the peak list from the chromatograms, data were processed, aligned and filtered using in-house software. Annotation of the content of the sample was accomplished by matching the extracted mass and retention time data from the chromatograms with a library of reference compounds and with a database of lipids.

### MS/MS lipid annotation

Chromatograms were recorded using data-dependent tandem mass spectrometry with full scan MS mode (Mass Range [100−1500]) and MS/MS in Top 3 mode using a normalised collision energy of 25. Acyl composition of DAGs and TAGs was established from the [M + H]^+^ precursor ion fragmentation with detection of [Acyl + NH4]^+^ neutral losses in positive ion mode with further combinatorial calculation of the acyl composition. Acyl composition of phosphoglycerolipids was established from the detection of [Acyl − H]^−^ fragments of the corresponding precursors in negative ion mode. Acyl composition of sphingolipids was established from the fragmentation pattern of [M + H]^+^ precursor ion in positive ionisation mode.

### GC–MS measurements, data processing and annotation

Samples were separated using a DB35 column (30 m × 0.25 mm × 0.25 μm; Agilent) with helium at 1.5 ml min^−1^ as carrier gas. Column temperature was initially held at 85°C for 2 min prior to the application of a linear temperature gradient of 15°C min^−1^ to a final temperature of 360°C. Data were exported to R from the Leco Pegasus software as NetCDF files and the Bioconductor package TargetSearch [84] was used to transform retention time to retention index (RI), to align the chromatograms, to extract the peaks, and to annotate them by comparing the spectra and the RI to the Fiehn Library [85] and to an in-house created library. Annotation of peaks was manually confirmed in Leco Pegasus. Analytes were quantified using a unique mass.

### Normalisation and statistical analysis

Normalisation was performed for each of the five analyses (GC, LC polar in positive and negative ion mode, LC non-polar in positive and negative ion mode) separately. The log_2_-transformed data were normalised to sample fresh weight and to the median intensity of groups where the median intensity from all features of a sample was subtracted from the intensity of each feature followed by the addition of the median calculated from the sample medians of the whole sample group as described in Rozema et al. [86]. Data of all platforms were merged to the final data matrix. One-way ANOVA was performed pair-wise between ‘In’ and ‘Out’ samples at all timepoints (*P*-value adjustment with Benjamini–Hochberg method [87].

## Data Availability

The findings of this study are supported by the data within the article and its supplementary materials. Additional information can be obtained by contacting the corresponding authors.

## Open Access

Open access for this article was enabled by the participation of University of Essex in an all-inclusive *Read & Publish* agreement with Portland Press and the Biochemical Society under a transformative agreement with JISC.

## Abbreviations

Cer: ceramide
DAG: diacylglycerol
dd-MS2: data-dependent tandem mass spectrometry
EI: electron impact
ESI: electrospray ionisation
FA: fatty acid
GC: gas chromatography
GlcCer: glucosylceramide
LC: liquid chromatography
lysoPC: (lyso)phosphatidylcholine
lysoPE: (lyso)phosphatidylethanolamine
MS: mass spectrometry
NCE: normalised collision energy
PC: phosphatidylcholine
PCA: principal component analysis
PE: phosphatidylethanolamine
PEG: polyethylen glycol
PG: phosphatidylglycerol
PI: phosphatidylinositol
PS: phosphatidylserine
RT: retention time
TAG: triacylglycerol
TOF: time of flight
UPLC: ultra performance liquid chromatography
