# Supplementary figures and images for: Chilling or chemical induction of dormancy release in blackcurrant (Ribes nigrum) buds is associated with characteristic shifts in metabolite profiles
Source: Biochem J. 2024 Aug 9;481(16):1057–73. doi: 10.1042/BCJ20240213 (PMC11346427; doi:10.1042/BCJ20240213)

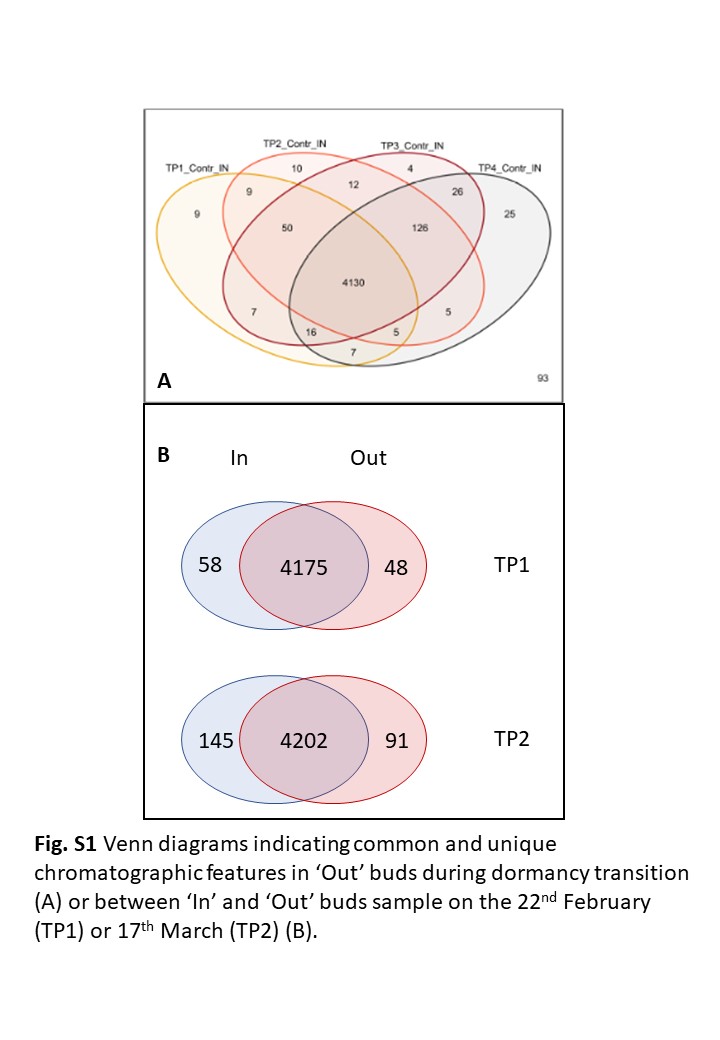

Supplement: Supplementary Material 1 [file BCJ-481-1057-s1.pdf]
